# Exploring two-step and one-step urgent care telephone triage: a UK-based semi-structured interview study

**DOI:** 10.3399/BJGPO.2024.0270

**Published:** 2025-12-19

**Authors:** Vanashree Sexton, Jeremy Dale, Carol Bryce, Helen Atherton

**Affiliations:** 1 Warwick Medical School, University of Warwick, Coventry, UK; 2 Primary Care Research Centre, University of Southampton, Southampton, UK

**Keywords:** qualitative research, triage, communication, primary health care

## Abstract

**Background:**

There are two models of urgent care telephone triage in the UK: one-step triage that is conducted by a clinician, and two-step triage involving an initial triage by a non-clinical call adviser followed by a secondary clinician triage. Both models may involve digital triage (computerised decision support). Little is known about patient experiences of receiving two-step triage.

**Aim:**

To explore patients’ and carers’ experiences of two-step triage in relation to experiences of one-step triage.

**Design & setting:**

Semi-structured interviews were conducted between July 2021 and February 2022 with patients and carers who had undergone one-step or two-step urgent care triage in England or Northern Ireland.

**Method:**

Data were thematically analysed; Oben’s conceptual framework of patient experience was used to interpret findings. Findings were reported in line with the Standards for Reporting Qualitative Research framework.

**Results:**

In total, 25 patients or carers were interviewed. Complexity, delays, and frustration were described in relation to two-step triage. Communication with non-clinicians was often experienced as scripted and inflexible, while communication with clinicians was described as more natural and empathetic. Reassurance experienced during triage enabled some patients to stay home without seeking further care.

**Conclusion:**

Minimising the complexity experienced by patients should be factored into planning two-step triage services. Further research should explore how digital triage can be adapted to promote more natural flow of communication and how empathetic communication during triage may enable self-care. Training for clinicians should emphasise such communication and the importance of giving sufficient time to patients during triage.

## How this fits in

Two-step urgent care triage, which is non-clinician led using a digital triage system, is intended to make best use of the clinical workforce. Our study explored patients’ and carers’ experiences of this model compared with one-step triage conducted directly by a clinician; to our knowledge, differences in experiences of these models have not previously been explored. Our findings highlight complexity and delays experienced by patients in two-step triage, and experiences of rigidity and unhelpful communication during non-clinician triage. Patient-centred and empathetic communication should be prioritised in the design of digital triage systems and in the training of both clinicians and non-clinicians who conduct triage.

## Introduction

Urgent care has been previously defined as *'the range of responses that health and care services provide to people who require — or who perceive the need for — urgent advice, treatment or diagnosis'*.^1^ Telephone triage plays a central role in the delivery of urgent care to patients. Internationally, triage typically follows a one-step model conducted by clinicians (usually nurses),^2^ for example, Healthdirect in Australia^3^ and the Ask Mayo Clinic telephone service in the US.^4^ In England, Scotland, and Wales, however, a two-step model has been adopted. Patients call the NHS 111 telephone service for urgent care advice, particularly outside of general practice opening hours (known as out of hours) and undergo an initial triage by a call adviser. Call advisers are non-clinicians (not medically trained); they are laypersons trained to provide advice to patients using a digital triage system (also referred to as computerised decision support) within the centralised NHS 111 service. About 50% of calls are transferred to urgent care.^5^

Two-step triage is intended to make best use of the clinical workforce. Two- step triage is intended to make best use of the clinical workforce, so given the increasing challenges in recruiting and retaining clinicians it is likely that this approach,^6^ which utilises non-clinician call handlers, may be used more frequently in future. While most of the UK population is covered by two-step urgent care triage, Northern Ireland (NI) has retained a one-step system whereby patients are typically triaged directly by a clinician.^7,8^ Hence, it is important to understand how this model affects patient experience.

Evidence to understand the two-step triage model is lacking. Benefits of telephone-based triage more broadly have included convenience in not needing to leave home and quick access to advice, compared with a GP appointment.^9^ The importance of nurses conducting triage having sufficient time in order for patients to feel reassured has been noted.^10,11^ Negative patient experiences have included the following: difficulty talking via telephone;^9^ needing to be assertive in order to receive the expected level of care;^10–13^ the number and relevance of triage questions;^9,14^ and staff being perceived to have *'poor local knowledge'* of nearby care providers.^9^

Understanding patient and carer experiences of the two-step (non-clinician-led) model and how this compares with one-step triage is important to designing patient-focused care systems. Importantly, patients’ experience of telephone triage may impact compliance with advice, potentially reducing its safety and effectiveness.^13,15^

We aimed to address the gap in evidence through investigating patients’ and carers’ experiences of two-step urgent care triage in England, comparing this to experiences of one-step triage in the NHS in NI.

## Method

We conducted a semi-structured interview study with patients and carers who had experienced telephone-based triage in urgent care in the UK. This study is reported in line with the Standards for Reporting Qualitative Research framework.^16^

### Recruitment and sampling

We aimed to recruit 25–30 participants, which was based on a previous study focused on the NHS 24 service in Scotland, and was expected to ensure data saturation.^9^

Patients and carers were recruited from two urgent care providers, in England and NI, which have different triage delivery models:

1. two-step triage (England), where most patients access clinician-led triage via the non-clinician-led NHS 111 telephone service; and
2. one-step triage (NI), where patients typically call the service, leave contact details with a receptionist, and are called back by a clinician who conducts triage.

The differing triage models allowed us to compare patient experiences within the NHS.

**Figure 1.**
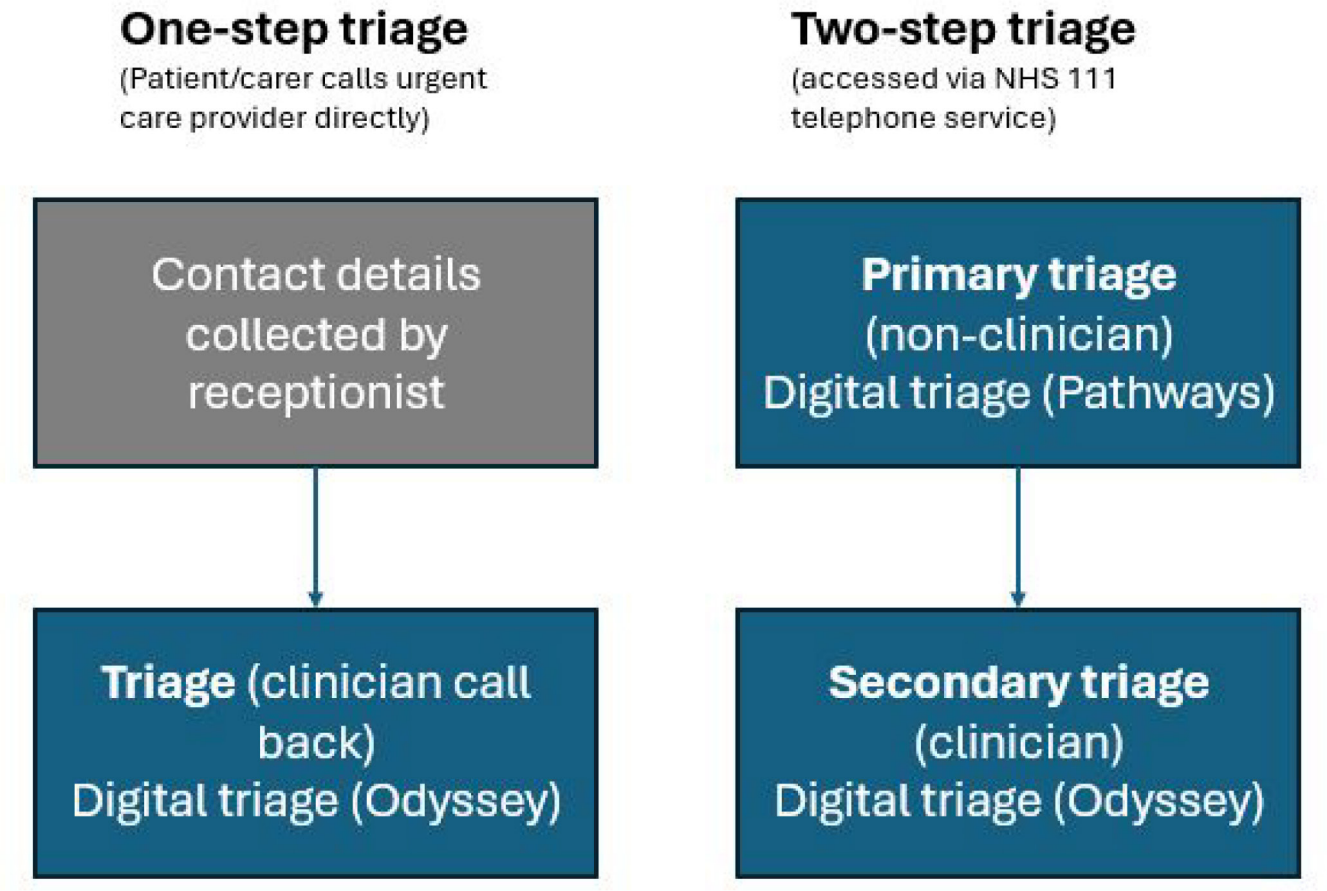
Visualisation of triage models.

Digital triage is used by both non-clinical and clinical call takers in England and NI to generate an urgency level, timeframe, and recommended care and/or referral advice for each patient. In England, non-clinician triage is conducted using the NHS Pathways algorithm.^17^ Clinician triage at both England and NI study sites was conducted using the advanced ‘Odyssey’ clinical decision support system.^18^ See Figure 1 for a visual representation of the two models.

A purposive sampling strategy was used to enable participant recruitment based on differing pre-defined characteristics relevant to the research aim.^19^

Service providers, who had access to patient demographics and urgency of triage advice, were instructed to invite participants based on quotas for: patient or carer, those calling on different days of the week, older age groups, ethnic minorities, and advice urgency level to include patients with low urgency advice (for example, self-care), as previous studies highlighted these patients may be less satisfied with services.^14^ Services invited consecutive callers within quota groups to avoid selection bias,^20^ where services may preferentially select patients who had positive care experiences. Participating services invited callers who had been triaged by a clinician in the past week to ensure that participants would be able to recall their experiences.

Participant eligibility (Table 1) was assessed by a senior clinician within the participating services. Callers meeting the eligibility criteria were informed about the study by the service; those expressing their interest in participating contacted the researcher (VS) via telephone or email.

**Table 1.** Eligibility criteria for potential participant selection

Inclusion criteria	Exclusion criteria
Participants (patients or carers) must: have been triaged or called on behalf of a patient who was triaged by the site in the past week; aged ≥18 years; not have any sensitive care problem; and have spoken English when calling the urgent care provider	Participants (patients or carers) were excluded if they were: aged <18 years; had a sensitive care problem (the following were excluded: calls relating to end of life, substance or alcohol misuse, suicidal thoughts, abnormal behaviour or thoughts, and complex social situations, such as domestic violence or where there may be safeguarding issues); unable to read and understand the information sheet and consent form; unable to understand verbal explanations in English; or did not speak English or had special communication needs (for example, use of translator)

### Data collection

Semi-structured interviews were conducted using open-ended questions, enabling experiences to be discussed in detail.

An interview guide was developed and piloted, building on guides used by the research team in similar studies.^21–23^ It was further refined following the first three interviews. Topics included accessing the telephone service, the triage call(s), and the overall care journey. See Supplementary Information S1 for the final guide used.

Before the interview, informed consent was obtained and the following participant characteristics were collected (as these could not be shared directly by the service providers before consent): 1) age group; 2) ethnicity; 3) postcode (deprivation, based on the Index of Multiple Deprivation,^24^ was derived from this); 4) the triage advice received from the clinician (for example, visit emergency department [ED] or self-care advice); and 5) approximate time and day of call. These characteristics provided contextual information for the analysis and were used to track participant characteristics as recruitment progressed.

Interviews were conducted via telephone or video-call, enabling participants located in different regions to take part. Interviews lasted between 30 minutes and 1 hour. Calls were securely recorded using Microsoft Teams and were transcribed by a professional transcription service (Appen). Transcripts were checked against audio files for accuracy.

### Analysis

Data were thematically analysed, reflecting the exploratory nature of the study. Six stages of thematic analysis were followed, according to the approach used by Braun and Clarke:^25^ 1) familiarisation, through listening to the recordings, and re-reading interview transcripts; 2) generating initial codes, which was completed using NVivo (version 11) software and was shared with and checked by two other researchers within the team. Coding and subsequent analysis stages were done iteratively in parallel with conducting interviews; 3) searching for themes, which was based on areas of similarity of codes; 4) reviewing potential themes, which was undertaken to ensure they fit the data; 5) defining and naming themes, which was refined through discussion; and 6) producing a report. These stages were completed iteratively with time for reflexivity built in.

The framework selected for interpreting findings was Oben’s conceptual framework of patient experience centred on the humanity of the unique individual patient, where their experience of healthcare services is multidimensional.^26^ Oben describes that factors influencing the patient experience include those relating to the person before the onset of disease including their physical, psychological, and social dimensions, such as family and community support.^26^ This framework supported us to consider the circumstances of the patient beyond the individual encounter and how this impacted on their experience.

### Research team

The study was designed by VS as part of doctoral research, with support from HA, CB, and JD. Interviews were conducted by VS; coding was completed by VS and thematic analysis conducted with HA and CB, with time built in to discuss reflexivity, including reflections of the researchers’ own experiences of urgent care and how the findings align with previous studies^21–23^ conducted by HA and CB.

### Patient and public involvement

A patient and public involvement panel was in place for the wider doctoral research, which this study formed part of. Feedback was sought during the design stage of this study, and feedback on the interview guide was incorporated; this included the ordering of the questions and prompts to ensure flow of discussion reflected the patient journey. Members were also invited to a dissemination event.

## Results

In total, 25 interviews were conducted between July 2021 and February 2022; 18 were England-based (using two-step triage) and seven NI-based (using one-step triage). More patients and carers were female (*n* = 17) than male (*n* = 8). Most were White British (*n* = 20), others were White Irish (*n* = 3), mixed ethnicity (*n* = 2), and Black African (*n* = 1). There were similar numbers of patients (*n* = 13) and carers (*n* = 12) and participants received a range of triage advice, from self-care to emergency service referral (see Supplementary Table S1).

The following three themes were identified: 1) complexity and delays; 2) communication barriers and facilitators (particularly in relation to non-clinician triage); and 3) influence of the patients’ confidence.

The impact of COVID-19 was identified as a cross-cutting theme and is reported here given the timeframe of the study. It provides context for some but not all of the findings.

COVID-19 had affected patients’ health-seeking behaviour; for example, some patients and carers expressed how the difficulty they faced in trying to get face-to-face care from their GP led them to contact 111 for the care they felt was needed:

*'“No, I'm not having no more of this" I said, "I want hands on, if you don’t get the paramedics out here soon, I’ll just take it further."'* (Partner of male patient, female [F], England, aged 65–74 years, White British, routine GP appointment; participant [P]3)

Frustration at the lack of communication from services about changes to the process (resulting from COVID-19) of accessing care was evident. Callers felt that services were not always upfront about their policies; for example, they described that face-to-face care was not available through the out-of-hours (OOH) service without a negative COVID-19 test, which was not made clear when first calling the service:

*'I always think if the information was made clear ... and if it was communicated to parents or to anybody calling them, that more or less, "Look, there’s no point, there’s no point", like, you know. Not there’s no point waiting on a call, but, "We would advise you to go A&E* [accident and emergency] *if you have Covid symptoms."*' (Mother of baby aged 12 weeks, F, NI, aged 25–34 years, White Irish, OOH doctor callback; P13)

### Complexity and delays

England-based participants shared their experiences of navigating a complex pathway, often involving several complicated steps. These were experienced as being time-consuming and that care was unnecessarily delayed. Although they expected and understood the need to speak with a non-clinician when calling the NHS 111 service they described delays in the process, including needing to wait for an additional callback from a clinician and needing to repeat information about their health problem in at least two different telephone calls. Some patients questioned why this was necessary:

*'I knew I just needed to get her antibiotics into her … knowing that I had to wait for another phone call and repeat it all again before anything would be done was a little bit frustrating … I'm not gonna lie, it is a bit of a pain repeating the whole, like, the whole thing over and over. … I don't know if that middle step* [clinician triage] *is totally necessary from, like, a patient point of view because I'm assuming the 111 caller’s* [call takers]*, I don't know, are they just as qualified to make that call to say, “Oh yeah, a GP needs to speak to you.” So is it totally necessary and efficient for that middle phone call?'* (Mother of child aged 3 years, F, England, aged 25–34 years, White Irish, triaged to OOH GP callback; P12)

Patients’ frustrations in relation to delays in two-step triage were compounded when they additionally experienced poor integration between services. For example, a patient who was referred in secondary triage to the emergency ambulance service described receiving a call from the emergency service, who referred her back to the initial triage at NHS 111 for a problem that subsequently required hospital admission. She expressed frustration and potential for clinical safety issues to arise from such delays:

*'So, had I been another hour messing about and 111 hadn’t got back to me or they hadn’t have realised that there was a big problem here ... and had it had been a different nurse, it could have been a different … So, it’s just, it was thanks to the nurse going, “This is ridiculous, the only other thing I can do is refer to the doctor and I’m gonna put it as urgent as I can.”'* (Patient, F, England, aged 25–34 years, White British, referred to emergency ambulance service; P11)

Navigating urgent care triage appeared to be a more complex process for patients in England, with patients expressing their frustration and concerns about receiving timely care owing to delays caused by needing to speak to different call takers and needing to describe symptoms repeatedly.

### Communication barriers and facilitators

Patients described rigidity in communication during triage, particularly non-clinician triage. They described it as repetitive and scripted or algorithmic, simply needing to provide ‘yes’ or ‘no’ answers and feeling unable to ask questions. In the quote below, the patient describes a lack of human touch in NHS 111 primary triage:

*'It’s all very synthetic and … I suppose it’s talking to a computer basically … rather than a human, you know? ... There wasn’t a lot of human touch. It was just tap, tap, tap. Now, next question. Tap, tap, tap.'* (Patient, male [M], England, aged 75–84 years, White British, triaged to routine GP appointment; P24)

Patients did not feel the rigidity of triage communication served any purpose in helping or supporting them. Clinician triage, however, was described as more natural, even when they acknowledged the use of scripted triage questions:

*'I mean, she, she may, she may have a set of questions that she has to ask … But she did it in a friendly manner, that it, it just felt like two people having a conversation.'* (Patient, M, England, aged 55–64 years, White British, triaged to routine GP appointment; P22)

This highlights the acceptability of digital triage being used during the telephone call, when it is not perceived to interfere with the natural flow of communication. Communication that was ‘reassuring’ was important to participants, particularly where self-care was being advised by the clinician; this was evident across both triage models:

*'So she* [triage nurse] *was very good, she said, “You need to write it down.” And then she gave me advice to put her head on a hot-water bottle and see if that would help ease her a bit, you know, it’s just, like, common sense really, but I didn’t think of it at the time … she’s very good, very reassuring, and that’s what I just needed at that time in the morning.'* (Mother of child aged 5 years, F, NI, aged 35–44 years, White British, routine GP appointment; P25)

Reassurance and empathy provided by the clinician helped in the patient’s decision to stay at home, rather than seeking further care, which they felt they may have done otherwise:

*'The nurse that phoned me back ... again, she was ever so thorough, she went through all different things with me to check on him. And she ultimately said, "Look if, if you really feel anxious I would go, just for you to put your mind at ease," she said, "I think he needs to be monitored. And if any other symptoms arise then, you know go to A&E." … Which ultimately I didn’t do ‘cause I thought, "You know what she’s right, perhaps it was me being a bit anxious." But she did calm me down a lot by kind of, reassuring me ... that you know, it wasn’t as bad as probably I thought it was.'* (Patient, F, England, aged 35–44 years, White British, triaged to self-care; P1)

Patients shared their appreciation for unhurried communication, being listened to, and having space to explain their symptoms during ‘natural’ conversations with a clinician, which contrasted with the rigidity that occurred with non-clinician triage. Below, a patient describes their positive interaction with a triage nurse:

*'You need someone to sit back and take time to listen … And that’s what, that’s what she did … She left it, she left the empty spaces that I could freely fill … you know, with relevant or non-relevant information, you know. And I think that’s how a nurse should be … a nurse or a doctor. They should do that. Because it’s not always what you say. It’s, it’s the information in between that can give clues on what’s going on.'* (Patient, M, England, aged 55–64 years, White British, triaged to routine GP appointment; P22)

Patients described difficulty in communicating their symptoms or feeling anxious, particularly when they felt the professional was under pressure and could not allocate enough time to listen or appeared not to take the problem seriously. Not being rushed was the antithesis of the rigidity sometimes experienced.

### Influence of the patient’s confidence

Many factors fed into the dynamic between patient and professional, including the patient’s confidence about the care they felt they needed, which was affected by support from family members, and fear of exposure to COVID-19 in subsequent in-person care. These factors influenced shared decision making and agreement of the triage decision.

Some patients described seeking validation and reassurance from a professional to help them make a judgement on the level of care that was required. They described a sense of being giving permission to access other services. For example, one patient reported a conversation with a paramedic conducting triage helped to confirm that care was needed, and that they would not be wasting the GP’s time:

*'Well, his advice was to see my GP straightaway and I, you know, as soon as the GP, as soon as I was able to call in the morning I, you know, I did that … And, and I mentioned the call that I’d had with the paramedic. And I think that probably just helped the GP understand that, you know, I needed some help and, and that I was following advice and, and not just, you know, wasting their time.'* (Patient, M, England, aged 45–54 years, White British, routine GP appointment; P6)

Another patient described feeling that the nurse helped to confirm the need to go into hospital:

*'She, kind of, confirmed my suspicions, I thought we should go to hospital.'* (Patient, F, England, aged 25–34 years, White British, triaged to emergency service; P11)

Participants needed to feel that their potential use of the health service had been validated by a healthcare professional. In contrast, others took agency and asserted their view on the level of care required. This was particularly evident for participants who were more familiar with their health problem, had family support, or owing to previous experiences of a long-term condition:

*'You know, if you haven’t, if you’ve been fortunate not to, you haven’t needed doctors and not to have been exposed to, you know, hospitals and things you mightn’t have the same … confidence, I suppose, in expressing yourself and knowing what these symptoms might be. I’m not saying I’m medically trained or anything like that, but, you know, my mum has had issues over the years.'* (Daughter calling about mother aged ≥75 years, F, NI, aged 45–54 years, White British, OOH GP callback; P20)

*'Well yes because we finally got the outcome that I wanted ... for my husband. And I suppose in one way it gave me a choice, at least I could voice my opinion.'* (Partner of male patient, F, England, aged 65–74 years, White British, triaged to emergency service; P3)

Considering Oben’s framework, which describes patient experience to be *'informed by a complex combination of the patient’s personal life, as well as their own and their family’s experiences within the health-care system at all levels of care'*,^26^ the two quotes above demonstrate that prior experience shapes the patient experience: for participant 21, their confidence related to a knowledge of a long-term health condition; while participant 3 asserted the care she felt was needed, this participant was a retired nurse and likely drew on this experience.

Evidently a range of different factors, including family support, knowledge, assertiveness, or need for validation influenced how patients communicated with call takers. These sometimes influenced the triage outcome (priority and type of referral) recommended at the end of triage.

## Discussion

### Summary

This study illustrates challenges faced by patients and carers in their use of two-step triage. Participants described feeling frustrated by the two-step model, including the need to answer questions repeatedly and the delays that this brought in gaining timely access to care. They described rigidity in communication, particularly in relation to non-clinician triage, which was perceived as being scripted and inflexible. In comparison, communication with clinicians was described as more empathetic, reassuring, and helpful.

There were areas of commonality in experiences of clinician triage in the two models. Patients appreciated the human element of communication, including reassurance and being listened to, which enabled some patients to feel confident to self-care at home. Such communication was experienced during clinician triage, but not in the non-clinician assessment.

Oben’s framework states: *'The patient experience, in essence, is the human experience of health-care services.’* ^26^ Through this holistic lens, we can understand patients’ poorer experiences of the rigidity in communication, which may result from call takers’ use of digital triage. In the context of this framework, the multifaceted nature of the patient experience, including factors outside of the immediate interaction with the service (for example, their anxiety in relation to their symptoms or their family member’s symptoms), also helped us to understand patients’ need for reassurance.

The framework further helps to understand the differing interactions influenced by the patient’s confidence, including down-playing of symptoms, which was described by some patients, and the potential vulnerability of those who do not have support of a family member, those who may be less assertive, or don’t have prior experiences of navigating health care.

### Strengths and limitations

To our knowledge, this is the first study to explore patients’ and carers’ experience of England’s two-step urgent care triage in comparison to one-step triage (deployed in NI).

A strength is that all the patients and carers were recruited across diverse settings and had been exposed to clinician triage using the same digital triage system; however, it is not possible to know whether their experiences would be the same with other digital triage systems. Similarly, experiences of non-clinician triage may be different according to different triage systems being used; however, similar experiences may be expected in relation to other heavily scripted yes or no binary algorithms.

Participating sites were asked to select consecutive callers based on a set of characteristics. This was intended to minimise self-selection bias compared with other recruitment approaches such as advertising directly to patients, and the study was successful in recruiting a broad range of patients and carers. However, fewer male and ethnic minority group participants were recruited, likely owing to response bias, with these groups typically less likely to participate in research. Information regarding ethnicity is not routinely available within the triage record; hence service providers had attempted to recruit ethnic minority group participants based on non-English patient names. Added to this, the study only included patients and carers who spoke English.

Our previous study showed lower call rates to these services among male patients;^15^ our lower recruitment of male participants means we have limited insight into experiences of male callers and how they may differ to female callers.

We additionally recruited lower numbers of participants from the Northern Irish service; however, this is not felt to have impacted on the study, as the aim was primarily to investigate experiences of two-step triage.

Another limitation is that data were collected in 2022 and so may have been affected by the pressures on healthcare systems during the COVID-19 pandemic. At this time services were under unusual pressure; however, many of the study findings are comparable with previous literature indicating that they were not pandemic specific.

### Comparison with existing literature

A previous study similarly reported a common area of dissatisfaction and delays in the process related to the initial triage questions.^9^ In contrast, greater use of open-ended questions by triage nurses may promote patient safety through allowing patients to express their concerns and checking patients’ understanding of triage recommendations.^27^ Ziebland *et al* ^28^ suggest clinicians require time to respond to and contextualise the complexity of the individual patient; understanding their symptoms may prevent re-consultation. This supports our finding that patients felt better able to manage their care needs at home when they felt the clinician conducting triage gave them sufficient time to discuss and explain their symptoms.

Our study highlights the importance of communication skills in delivering digitally supported triage. Empathy experienced during patient–clinician communication is known to be important to patients and has been reported to have a positive impact on patient outcomes in a range of healthcare settings.^29,30^ This echoes our findings in relation to clinician triage and suggests the need for empathetic communication to be better incorporated into all types of urgent care triage.

Patients described that reassurance and empathy helped them to feel confident in managing their health concern, in some cases enabling them to self-care and stay at home. The importance of reassurance has been previously reported in urgent^31^ and emergency care^32,33^ settings, where anxiety may drive some patients to use services when they are seeking reassurance rather than needing in-person care.^32,33^

### Implications for research and practice

The complexity, delays, and duplication involved in two-step urgent care triage experienced by patients should be taken into consideration when planning non-clinician-led services. Measures could be taken to help make processes clearer for patients; for example, non-clinician call takers could explain their role and the further triage process more clearly. Further research should explore how digital triage, particularly when used by non-clinicians, can be designed to promote more natural flow of communication, to enable information flow and avoid repetition of questioning. Service commissioners and providers should ensure sufficient time is allocated to allow patients’ needs to be sufficiently considered and discussed. Call-taker training should emphasise the importance of reassurance and sufficient time given to patients during triage. There is a need for research to further explore the impact of such communication on enabling self-care where appropriate, which may help to avoid unnecessary subsequent use of other healthcare services such as EDs.

In conclusion, this study highlights that the two-step urgent care triage model is experienced as introducing complexity and delays to patients and carers, who found it to be time-consuming and frustrating because of the need to provide the same information repeatedly. We highlight factors that facilitate communication during triage, including the importance of empathetic and reassuring communication, and ensuring sufficient time for triage assessments; further research is needed to explore how digital triage systems and training can be designed to encourage these human elements of communication.
